# Avian Leukosis: Will We Be Able to Get Rid of It?

**DOI:** 10.3390/ani13142358

**Published:** 2023-07-19

**Authors:** Sergio Fandiño, Esperanza Gomez-Lucia, Laura Benítez, Ana Doménech

**Affiliations:** 1 Department of Animal Health, Veterinary Faculty, Complutense University of Madrid, Av. Puerta de Hierro s/n, 28040 Madrid, Spain; sergifan@ucm.es (S.F.); domenech@ucm.es (A.D.); 2 Department of Genetics, Physiology and Microbiology, Faculty of Biological Sciences, Complutense University of Madrid (UCM), C. de José Antonio Novais 12, 28040 Madrid, Spain; lbenitez@ucm.es; 3 Research Group, “Animal Viruses” of Complutense University of Madrid, 28040 Madrid, Spain

**Keywords:** avian leukosis virus (ALV), ALV-J, ALV-E, recombination, chicken, wild birds, resistance, cell receptor

## Abstract

**Simple Summary:**

Historically avian leukosis has produced severe economic losses, due to decreased productivity, higher mortality from immunosuppression-associated infections, and tumor development. It is virtually eradicated in commercial poultry of the western world, but infection by avian leukosis virus (ALV) still remains in hobby, fancy, backyard and native and indigenous chickens from which it may jump to commercial birds. In addition, relics of ancient infections by ALV remain in the genome of birds, with which ALV may recombine and generate new viruses. In this review we analyze the virus, its infection in the cell, and how the immune system of the bird reacts against it to better understand the strategies which are being explored to avoid the reemergence of this disease. Scientific research focuses on manipulating the genome of birds, in order to obtain animals that the virus cannot infect, due to the absence of a compatible receptor in the cells, or modify their immune response to make it stronger. In addition, hens and chickens are analyzed to eliminate those which might be infected. But, more studies are needed to better understand this complex disease.

**Abstract:**

Avian leukosis viruses (ALVs) have been virtually eradicated from commercial poultry. However, some niches remain as pockets from which this group of viruses may reemerge and induce economic losses. Such is the case of fancy, hobby, backyard chickens and indigenous or native breeds, which are not as strictly inspected as commercial poultry and which have been found to harbor ALVs. In addition, the genome of both poultry and of several gamebird species contain endogenous retroviral sequences. Circumstances that support keeping up surveillance include the detection of several ALV natural recombinants between exogenous and endogenous ALV-related sequences which, combined with the well-known ability of retroviruses to mutate, facilitate the emergence of escape mutants. The subgroup most prevalent nowadays, ALV-J, has emerged as a multi-recombinant which uses a different receptor from the previously known subgroups, greatly increasing its cell tropism and pathogenicity and making it more transmissible. In this review we describe the ALVs, their different subgroups and which receptor they use to infect the cell, their routes of transmission and their presence in different bird collectivities, and the immune response against them. We analyze the different systems to control them, from vaccination to the progress made editing the bird genome to generate mutated ALV receptors or selecting certain haplotypes.

## 1. Introduction

Avian leukosis virus (ALV) is an alpharetrovirus associated with tumorigenic disease, decreased fertility (including a drop in egg production) and growth retardation [1,2,3]. In addition, this agent causes severe immunosuppression, increasing susceptibility to other microbial infections and the risk of failure in subsequent vaccinations against other diseases [4]. ALV is not a single virus, but a variety of viruses, which are closely related to Rous sarcoma virus (RSV) and thus are sometimes referred to as Avian Sarcoma and Leukosis Virus (ASLV). In many instances the differentiation between both viruses is non-existent and authors include tables containing both ALV and RSV sequences as “ALV” or report RSV sequences under the name “ALV”. ALV has also been used to refer to other alpharetroviruses carrying oncogenes on the ALV species sequence backbone. Thus, ALV can be considered “sensu lato” (all ALV-related viruses found in birds) and “sensu stricto” (only slow transforming ALV). In this review we will focus mainly on ALV “sensu stricto”, which do not induce fast cell transformation, in contrast to RSV and other related viruses. ALVs cause disease derived from chronic infection and integration, and may sometimes activate an oncogene to cause disease. Throughout history avian leukosis has produced high economic losses and creative measures have been implemented to decrease its impact, including the development of ALV-resistant chicken breeds. In this review we analyze the virus and the disease it produces to better understand whether it will be possible to reduce its presence and effects.

### 1.1. Avian Sarcoma/Leukosis Virus (ASLV) in the Past and in the Present

#### 1.1.1. ASLV in History

First mentions of what could have been ASLV infections date from the 19th century by Roloff in 1868 and Caparini in 1896 [5]. Detection of osteopetrosis in chickens remains from the Roman empire period has also been attributed to ASLV [5]. Early research on ALVs was carried out in the 1900s when viruses were still mostly unknown. In 1908 Ellermann and Bang (1908) reported that certain chicken leukemias were transmissible using filtered blood [6], and in 1911 Rous demonstrated that chicken sarcoma could be transmitted using cell-free extracts [7]. With this, he set the scenario for the discovery of the virus that bears his name, Rous sarcoma virus (RSV), for which he received the Nobel Prize in 1966. In the beginning ALV was also named as Rous-Associated Virus (RAV; [8]).

The importance of these viruses in molecular biology has been enormous. In 1958, Rubin and Temin observed changes in chicken fibroblast morphology following exposition to a chicken sarcoma extract [9], and cell aggregation that could represent the start of tumorigenesis. It would be later known that this was the effect of the *src* oncogene, a gene present in RSV which induces fast transformation, and which opened the discovery of several more oncogenes and a better understanding of oncogenesis [10]. Later, Temin [11] and Baltimore [12], working independently, would be the first proponents of DNA integration by retroviruses and would discover the first reverse transcriptase in their works with RSV and with murine leukemia virus, respectively.

Besides the undoubtedly crucial contributions of the ASLV group to molecular biology mentioned in the previous paragraph, another relevant contribution has been the discovery of defectiveness in viral replication, first discovered in the Bryan Hightitter RSV strain [10]. Later work in the late 60s with this strain led to the discovery that some cells had helper activity conferred by endogenous retroviruses, which will be discussed later in this review. Other less-studied ASLVs were discovered in the first half of the 20th century, including the avian erythroblastosis virus by Engelbreth-Holm and Rothe Meyer [13] or the avian myelocytomatosis virus, MC29 [14].

#### 1.1.2. Current Relevance of ALV

As mentioned above, there are two types of ASLVs: fast- and slow-transforming ASLVs. Fast-transforming viruses carry an oncogene and all of them (except most strains of RSV) are defective, requiring the presence of a helper virus to complete their replication cycle. They induce the development of tumors in a wide variety of organs of chickens in less than three months after initial exposure, causing their early death. Slow-transforming ALVs (ALV “sensu stricto” and the object of this review), which are replication-competent, produce tumors in only a small percentage of chickens [15].

Since its discovery it has been one of the most studied retroviruses to date, and nowadays ALV remains one of the highest potential pathogens in the poultry industry. ALV is almost eradicated in the western world in commercial chicken breeds [16,17], but in China ALVs still represent a problem because they persist in many native chicken breeds, causing important economic losses in production [18,19]. Surprisingly, a recent study has found that the infection may be present in up to 28.7% of the fancy chickens sampled in Saxony (Germany) and up to 56.0% of their flocks [20]. Moreover, because the Chinese poultry industry is less organized, especially among the local breeds of chickens, ALV will exist for a long time with a potential danger for the rest of the world [21,22]. Economic losses are estimated in the range of millions of US dollars each year [5].

To date there are 11 subgroups of ALV, named A to K (see below). Traditionally, ALV-A followed by ALV-B have been the most prevalent subgroups in western countries, although with a low incidence of clinical neoplastic disease (1–2%) associated to them. It is estimated that, when ALV circulates within a flock, mortality rates usually range between 1 and 2%.

Chickens afflicted by ALV present nonspecific signs such as weakness, loss of appetite, diarrhea, dehydration and thinning. Other signs are not as apparent, including anemia, immunosuppression and hepatitis. Some chickens may die without showing signs of disease, and economic losses can be up to 20% associated to subclinical pathologies, growth retardation and declination in production performance, decreased egg production or quality, decreased hatchability, later sexual maturation and immunosuppression [1,5,21], associated with 5–15% increased mortality due to other causes [5].

The infection may affect the circulatory system in the form of myocarditis and chronic circulatory syndrome as described after RAV-1 ALV-A strain inoculation [23]. It has been reported that RAV-7 ALV-C strain may induce central nervous system affectation in the form of ataxia, lethargy and imbalance [5]. Occasionally ALV-infected chickens present tumors. The oncogenesis specificity of ALV is dependent on LTR and *env* sequences and it is different between viruses in the same subgroup [24]. In the oncogenic process, not only the promoter in 5′ LTR but also in 3′ LTR may initiate the transcription of potent cellular oncogenes [25].

The possible presence of ALV in vaccines has raised alarm. Although ALV reverse transcriptase and nucleic acids were found present in several human commercial vaccines grown from chicken cells, human transmission has not been reported and it is currently accepted that ALV and other chicken oncogenic viruses cannot be transmitted to humans or adapt to mammalian hosts [26,27,28,29]. Thus, it is the general consensus that chicken-derived vaccines are safe for humans. The fact that ALV may contaminate veterinary vaccines is a more pressing concern (e.g., Marek’s disease: [30]; Newcastle disease: [31]), as they have been shown to induce pathogenicity in commercial three-yellow chickens in China [31]. Though Mao et al. [32] found only three veterinary vaccine batches out of 918 contaminated with ALV, ALV contamination of vaccines could be a transmission route of ALV to commercial chickens [31].

## 2. Transmission of ALV

### 2.1. Routes of Transmission: Horizontal Versus Vertical

**Figure 1:**
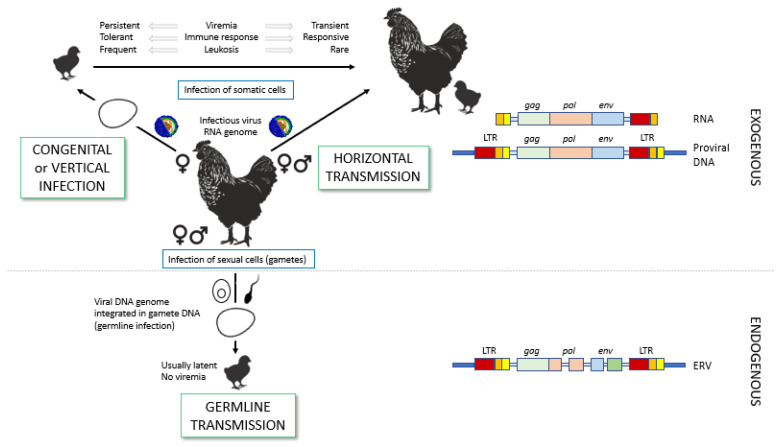
Transmission routes of ALVs, and differences between exogenous and endogenous ALVs. Adult birds can transmit the virus through the egg, generally considered as vertical transmission; the consequences are different depending on whether it is in the albumen or yolk (congenital transmission) or through the germline. The three different states of the viral genome are shown: RNA in the viral particle, proviral DNA in the somatic cell (characterized by the presence of LTRs flanking the provirus), and endogenous retrovirus (ERV) in the germline (which usually do not express functional proteins) (adapted from [1]).

A well-known characteristic of the *Retroviridae* is their ability to become endogenized, when they successfully infect germ cells and are transmitted to the ensuing generations according to Mendelian laws. This is the case of ALVs, which contain both exogenous and endogenous members. Exogenous ASLVs are transmitted horizontally from one individual to another or vertically (congenitally) from progenitor to offspring prior to birth (Figure 1). Infected chickens shed viral particles in their fluids, mainly feces, and horizontal transmission can be direct or indirect through contaminated fomites [33]. Hens can transfer both viruses and specific antibodies to their progeny. ALV is shed into the egg albumen as a consequence of virus production by albumen-secreting glands of the oviduct, although not all eggs that have ALV in the albumen give rise to infected embryos or chicks because of neutralization of the virus by maternal antibodies in the yolk [5]. Congenital infection of chickens infected prior to hatching is rare, but it is relevant because congenitally infected chickens can act as horizontal infection sources and it ensures the continuity of the virus within a population even in low prevalence. These chickens develop immunologic tolerance to ALV due to early exposure [34]. The male parental contribution through ALV-infected semen to congenital infection was shown by Li et al. [35] by demonstrating that chicks born from hens infected through artificial insemination with ALV-J-containing semen could be infected by this virus. This was seen in 1 of 34 chicks born, so the event must be rare and probably plays a negligible role in field conditions. As mentioned, maternal antibodies in the egg are protective, and the susceptibility of chickens to horizontal infection increases in the absence of maternal antibodies and the presence of endogenous retroviruses, especially those associated with the slow-feathering sex-linked K gene [36].

**Figure 2:**
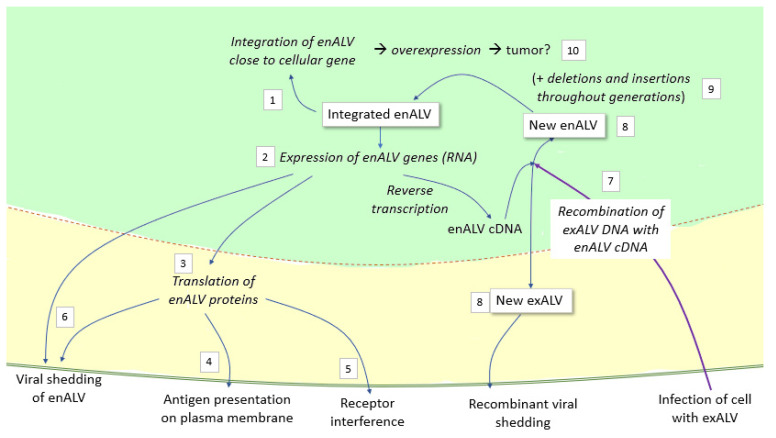
Generation and impacts of endogenous retroviruses. When an exogenous ALV (exALV) infects a sexual cell, it can become endogenous (enALV) and stay integrated in the cell genome (germline transmission) (1). Occasionally RNA can be transcribed (2) and translated in the cytoplasm (3). enALV proteins can be presented on the cell surface as antigens (4), interfere with exALV by blocking the receptors (5), or, along with enALV RNA, assemble into new viruses to be shed from the cell (6). When the cell is infected by an exALV, the resulting DNA may recombine with enALV cDNA (7) or with proviral enALV in the bird genome and give rise to new proviruses (8). If the new provirus suffers deletions or insertions, it will become defective (9). When enALV integrates in the proximity of a cellular gene, the potent promoters in the 3′ LTR may control its expression (10). Cellular processes are shown in italics, the nucleus in light green and the cytoplasm in cream (adapted from [38]).

As other retroviruses, ALV may be also transmitted in the germline throughout generations, becoming endogenous (Figure 1). Many of the endogenous ALVs (most of them of subgroup ALV-E) are defective and their virions are non-infective. However, their contribution to ALV pathogenesis may be relevant. Their genomic structure is similar to their exogenous counterparts, containing the same basic genes, though some of them may be incomplete due to deletions or insertions throughout the generations. Due to homologous LTR recombination (the repeated sequences flanking the DNA proviral genome, described in Section 3.1), in some cases deletions may go as far as leaving only lone LTRs (solo LTRs) [37]. Though this may render them incompetent in most cases, these proviruses or remnants of proviruses may be expressed and translated, and the cell may exhibit translated proteins on their membrane. Env and mRNA encoded by ALV-E may also interfere with recognition of exogenous ALV and be protective to the chicken, or viral shedding may happen, either of ALV-E particles or of ALV recombinants, displaying parts of the genome of ALV-E and parts of other ALVs (Figure 2).

### 2.2. ALV Classification into Subgroups

Based on the host range, the amino acid sequence of the envelope glycoprotein, gp85(SU), the receptor usage patterns in susceptible and resistant avian cells and the molecular biological characteristics of the viral genome, including whether they are exogenous or endogenous, the ALV family members are divided into 11 subgroups, ALV-A through ALV-K (Table 1). Subgroups A, B, E, and J are the most studied [1,21,39]. Simultaneous infection with more than one subgroup occurs very rarely. Fenton et al. [40] described the circulation of both ALV-J and ALV-A in broiler breeder flocks in Australia, and even the detection of dual infections in the same chicken. This concurrent presence of more than one ALV subgroup is of particular concern because it would represent an opportunity for these viruses to recombine and extend their host range. However, though dual infections are rare, recombinants are frequent and are one of the most concerning issues in understanding ALV biology.

**Figure 3:**
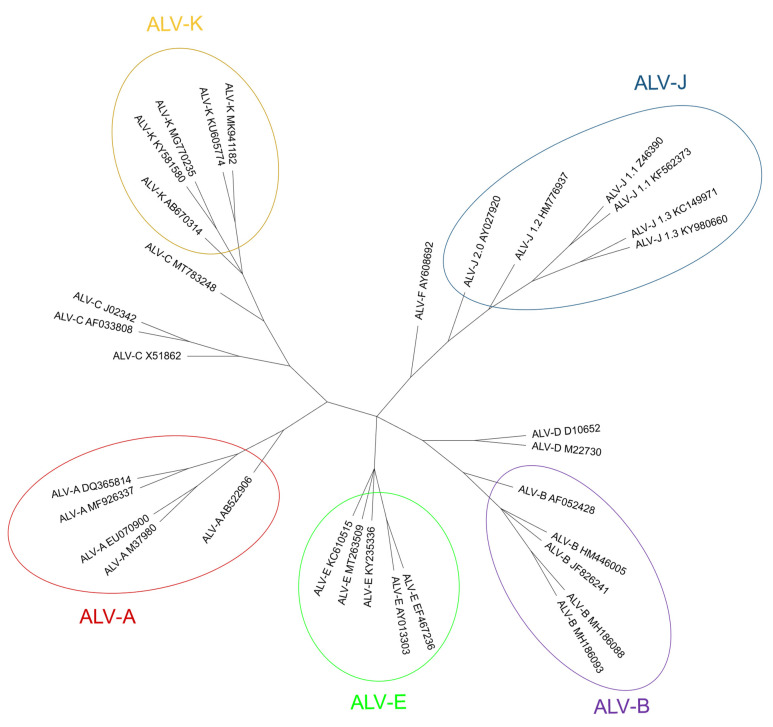
Maximum-likelihood phylogenetic inference of 33 gp85(SU) amino acid sequences of ALV from different subgroups. The tree was inferred under a JTT matrix-based model with gamma correction 0.68 based on 1000 boots. Branches reproduced in less than 50% of the replicates have been collapsed. The tree was created using MegaX and edited in iTOL.

A phylogenetic tree of the different ALV subgroups with published sequences in GenBank, including ALV-J sub-lineages (1.1 to 2, identified by [41]) is shown in Figure 3.

#### 2.2.1. Exogenous ALV Subgroups

ALV subgroups A, B, C, D, J and K are exogenous. From these, ALV-A and ALV-J are the most prevalent while the others are scarce in comparison [15,42], though ALV-K and recombinants are not rare in some populations [20,43]. ALV-A and ALV-B are recognized as the classic and common pathogenic exogenous viruses that induce lymphoid leukosis, erythroblastosis [44,45], as well as glioma development [46] in chickens. Subgroups C and D have been reported rarely in the field (reviewed in [47]), and there is little research in which they are characterized, besides the receptor they use for cell entry, quite opposite to the situation of ALV-J, which has received the most attention in recent years.

ALV-J was first isolated in 1988 in broilers as a virus producing myelocytic myeloid leukosis in England [48], though no field cases of ALV-J infection or tumors in layer chickens were observed worldwide until 2004 [49]. Differently from A and B subgroups, ALV-J mostly induces myeloid leukosis in the host in the form of myeloblastosis, myelocytomatosis [1,15] and hemangioma [50]. It has originated from recombination between exogenous retroviruses and the EAV-HP retrotransposon from the EAV family (other endogenous sequences different from ALV-E) to which they present a similarity of 97% for the *env* gene [1,51], compared to only 40% to the gp85(SU) sequence of ALV-A through ALV-E [5,52]. It has several molecular particularities in comparison with other subgroups. One of them is the presence of the E-element which is a stem-loop in the 3′ untranslated region that is involved in viral infectivity and in oncogenesis in certain lines of chickens [24]. This element is homologous to the exogenous virus-specific region (XSR) present in certain Rous sarcoma virus strains [24] which has been characterized as a carcinogenic micro RNA (miRNA) [53]. In addition, gp85(SU) of ALV-J is highly variable, even in the same bird, as up to 5.1% divergence between gp85(SU) DNA was found in sequences isolated from different organs in the same individual [54,55]. The amino acid identity between individuals may be as low as 86.2% [55] and four [41] or five [56] different clusters of ALV-J may coexist. This high variability makes the use of a universal PCR protocol for the detection of ALV-J difficult [55] and has biological consequences, including the possibility of escaping to the immune response.

ALV-K is the most recently characterized subgroup. It was first identified in South China in 2012 in yellow broilers and it was determined that it caused a milder disease than other subgroups [57], which may be due to decreased LTR promoter activity [58] or a weaker binding capacity of ALV-K gp85(SU) to its receptor, Tva [59]. Nucleotide sequence analysis of viruses in this subgroup shows that the *gag* and *pol* genes have high sequence similarity (>94%) to those of ALV-A through ALV-E and ALV-J (especially to those of ALV-E, 96%) [58], but may be as low as 44% for ALV-J gp37(TM), contrarily to gp85(SU), which shows low sequence similarity with that of other subgroups (<87% to ALV-A to E and <20% to ALV-J) [58,60].

#### 2.2.2. Endogenous ALV Subgroup

Endogenous retroviruses (ERV) represent approximately 3% of the chicken genome [61], and in ALV are mainly represented by ALV-E, though other subgroups are considered in other bird species (Section 4.5). ALV-E, which is greatly homologous to the exogenous ALVs, was the first endogenous retrovirus identified in chickens [62]. It is located at various segregating *loci* in the genome [21], the *ev* loci, and initially endogenous ALVs were called *ev* genes. ALV-Es exist in many different sizes, ranging from full length proviruses to those with partial deletions, even leaving solo LTRs [63]. The different ALV-Es are typically present in low copy numbers in the genome. Of all *ev* genes, *ev21* may be expressed as a full-length infectious endogenous ALV, EV21, which can produce sometimes fully infectious viral particles [36,64]. It is one of the most relevant because it is related to the sex-linked K gene, expressed only in hens, which may transmit it congenitally to the progeny. It was considered to regulate slow feathering, but nowadays it is also associated with low productivity [36,65,66,67]. Though devoid of possible pathogenic ability by itself, ALV-E can also boost the incidence of certain lymphomas when present in coinfections with the Marek disease virus, including attenuated vaccine viruses [68], or even to reactivate otherwise-silenced ALV-E in the genome and increase the incidence of spontaneous lymphoid tumors [69]. It has even been reported that ALV-E may be associated with the development of ovarian adenocarcinoma in older hens [70], and the reduction in productivity traits, such as growth rate and body weight in broilers [71,72,73,74,75], as well as albumen height, egg weight, egg production, and sexual maturity in laying hens [71,72,73]. 

Although ALV-E could be typically negatively associated with productivity in a commercial setting, it also has positive effects in poultry. ALV-E infection induces immune tolerance to exogenous ALVs, but it also interferes with exogenous infection using several mechanisms such as receptor interference due to the expression of ALV-E *env*, or to the regulation of host *loci* with effector functions (Figure 2) (reviewed in [38,76,77]), and the inhibitory action of an anti-sense long non-coding RNA (lncRNA) [78]. Their potential role in defense against exogenous ALV may provide an overall net benefit in the productivity of indigenous chickens [38]. Due to its possible beneficial or detrimental effects, research on the characterization of ALV-E and its diversity in chickens, and possibly in other Galliformes, has increased in recent years. Recent in-depth studies have shown the great diversity present across chicken populations, with more than 400 new different ALV-E insertions described in some studies [38,79,80,81]. The current total of computationally predicted ALV-E insertion sites well exceeds 1300 sites in the chicken genome [38]. A list of all validated ALV-E loci to date is shown in Supplementary Table S1.

**Figure 4:**
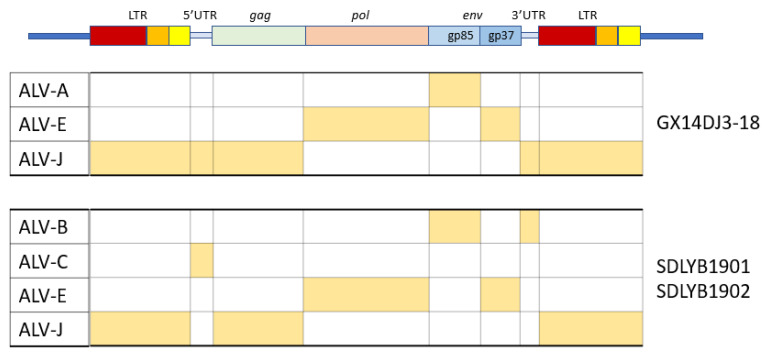
Schematic representation of the parental ALV subgroups to which recombinant isolates GX14DJ3-18 [87], SDLYB1901 and SDLYB1902 [83] show the highest sequence similarity. UTR, untranslated region. LTR, long terminal repeats.

ALV-Es may retain some structural integrity facilitating persistent retroviral gene expression and recombination with other endogenous or exogenous retroviruses [63], contributing to increase the pathogenesis of other subgroups,. It has been shown that natural recombinant ALV viruses containing ALV-E and parts of the genome of other subgroups (Figure 4) may be present in many different isolates, as well as in contaminated commercial vaccines. For example, two novel ALVs were isolated from domestic chicken breeds in China, whose *env* genes were uniquely different from the *env* of ALV-A through ALV-J, and carried an LTR cluster from ALV-E [82] or a novel ALV-K virus identified in a local Chinese yellow broiler in China that probably arose by recombination of ALV-K with endogenous ALV-E [58]. In other studies, other novel ALVs recovered from chickens with myeloid leukosis or which were asymptomatic were found to be recombinants between the different subgroups [83,84,85]. In addition, three natural recombinant ALV expressing ALV-A gp85SU, and ALV-E LTRs were isolated from contaminated commercial Marek’s Disease vaccines [86]. All these data suggest that ALV-E plays an important role in generating new ALVs, serving as a genomic pool for the development of recombinant ALVs and that this event is not infrequent, generating variations and potentially new genes [5]. The presence of ALV-C is notable in some of these recombinants, a subgroup which is rarely found in field cases.

## 3. The Avian Leukosis Virus, Its Characteristics and Properties

ALV, along with RSV and other similar viruses, is a member of the *Retroviridae* family, belonging to the *Alpharetrovirus* genus. ALV virions are enveloped and have a 35–45 nm in diameter capsid which has C-type morphology. The total size of the virion is between 80 and 120 nm [88,89]. They are enveloped viruses which exhibit glycoproteic spikes inserted in the envelope, which has a rounded (knobbed) shape [5].

### 3.1. The ALV Genome

**Figure 5:**
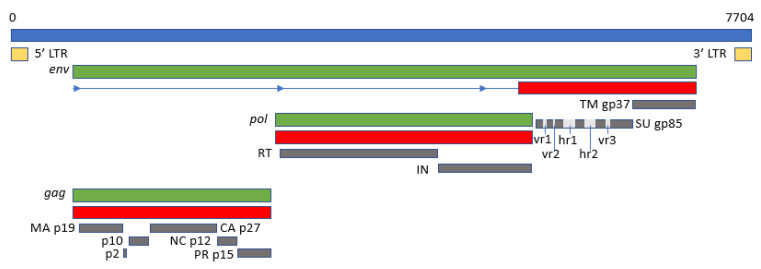
Schematic representation of the genome of ALV KU375453 (ALV-A), adapted from the GenBank graphics feature. The blue box represents the complete genome and its length in base pairs (bp); green boxes represent ORFs; red boxes represent polyproteins; grey boxes represent mature proteins; light grey boxes represent domains in SU related to receptor interaction; cream boxes represent the LTRs. The position of hr1, hr2, vr1, vr2 and vr3 is adapted from [90]. MA, matrix; NC, nucleocapsid; CA, capsid; PR, protease; RT, reverse transcriptase; IN, integrase; SU, surface; TM, transmembrane.

The viral genome is around 7.2–7.8 kb in size and has an organization consisting of *gag-pro-pol-env* (Figure 5), which is standard for simple retroviruses. The gene *gag-pro* encodes the inner proteins, including the capsid protein, p27(CA), an antigen common to all subgroups of ALV [20] as well as the matrix protein (MA), p10, nucleocapsid protein (NC), and the protease (PRO). As in other retroviruses, *pol* encodes the proteins necessary for viral replication [5].

Gene *env* (envelope) encodes the surface (SU, gp85) and transmembrane (TM, gp37) proteins, which are cleaved from the Env precursor trimer during maturation. gp85 (SU) binds to the cell receptor in a two-step process with intermediate structures. This unusual mechanism has allowed characterization of the binding steps [39]. gp37 (TM), a transmembrane glycoprotein, anchors gp85 (SU) to the viral envelope and has fusogenic properties [91,92]. Env, besides LTR, is highly determinant of the type of tumors chickens will develop, either lymphoid or erythroid, or even osteopetrosis [93]. The receptor specificity of gp85 (SU) permits the classification of ALVs into the subgroups mentioned above according to the sequence of two hypervariable regions, host range region (hr)1 and hr2, and three less variable regions, variable region (vr)1, vr2 and vr3 (Figure 5). It is highly immunogenic and determines the production of neutralizing antibodies. ALV from the different subgroups tends not to cross-neutralize, except for partial cross-neutralization between subgroups B and D [94,95]. A variety of studies have identified hr1 (aa194–198 and aa206–216) and hr2 (aa251–256 and aa269–280) as the main binding domains between the viral gp85 (SU) and the host protein receptor [59,96], with vr3 contributing to the specificity of the receptor interaction for initiating efficient infection [97]. Thus, polymorphisms in hr1 and hr2 regions may determine susceptibility due to increased or decreased binding capability, with the possibility that different or new subgroups of the virus arise if the hr regions bind to a different chicken cell surface protein [21]. Since the variability of hr regions in circulating ALVs establishes cell receptor binding, this knowledge may be useful for determining cell receptor edition or selection strategies.

The proviral genome (DNA genome integrated in the host chromosome) is flanked by identical sequences known as the Long Terminal Repeats (LTR), which have three functionally different regions which, from upstream to downstream, are: U3 (contains enhancer elements and the promoter), R (start of transcription) and U5 (with post-translation regulatory elements and a role in polyadenylation) [98,99] and a direct ribosome binding site (IRES), which enables cap-independent translation [100]. As in other retroviruses, U3 in the 5′ LTR is the controlling element of transcription because it contains transcription binding sites (TBS) which react to cellular signals enhancing transcription [101,102]. Some of them are AP-1, c-ets, STAT5, NF-1, or a glucocorticoid receptor. With the exception of AP-1, which is only present in ALV-E, the rest are equally present in other sequences studied. A large number of C/EBP predicted binding sites are detected in the U3 of both ALV-A and ALV-E GenBank sequences [103]. Most of the TBS are involved in the response to interferon (IFN)-α/β, IFN-γ, interleukins (IL), toll-like receptors (TLR) and other pattern recognition receptors (PRR), or are involved in cell proliferation or inflammation [104,105,106,107,108,109]. Interestingly, LTRs from endogenous ALV-E proviruses in the chicken genome have a very different sequence motif when compared with exogenous subgroups LTR sequences, resulting in different TBS which probably affect the response to cell factors [103]. Contrasting with U3′s high variability, U5 is mostly conserved among all ALV subgroups [103].

### 3.2. ALV Infection of the Cell

The development of tumors in slow transforming ALVs greatly depends on the integration of the provirus in the vicinity of cellular genes involved in cell growth and differentiation. ALV has a preference for integrating near expressed or spliced genes, at least in cultured cells [110] but, in most cases, integration can be described as random and unpredictable. Because it is random, the provirus is often inserted near a gene *locus*, directing its expression. ALV oncogenesis starts when an oncogene like *c-myc*, *c-myb* or *c-bic* is up-regulated by the potent enhancer elements in the 5′ LTR [5]. This is termed as insertional oncogenesis [15,111]. Curiously, *c-bic* was later found to encode a microRNA, miR-155 [112].

Thus, the integration site determines the progression of the disease. This is the case of the clonal progression of B-cell lymphomas in chickens infected by ALV [110,113]. The *MET* gene is a common integration place in hemangiomas induced by ALV-J [114]. This gene encodes a well-studied receptor of tyrosine kinase that binds hepatocyte growth factor/scatter factor (HGF/SF) and plays important roles in normal development and in a wide range of human cancers [114]. In an individual ALV tumor, between 700 and 3000 novel integration sites can be observed, with an average of 2.4 to 4 successful integrations per cell [110] and a maximum of 20 integrations reported in a single cell [5]. 

ALVs have been classified into noncytopathic (subgroups A, C, and E) and cytopathic (subgroups B and D) viruses depending on whether they induce cytotoxicity in cultures of avian cells. ALV-J, though quite pathogenic in vivo, fails to produce a cytopathic effect in cell culture [115]. Under certain circumstances, ALV-C has been observed to cause cytotoxicity in certain avian cells [39]. ALV-E has also been shown to induce cell death in experiments using quail and turkey cells [116]. The ALV-induced cytotoxicity is a slowing of cell division with the rounding and release of dead cells from the matrix, rather than a fusion of multiple cells to form syncytia [39].

## 4. ALV Is Not Only Present in Chickens

### 4.1. Host Range

Chickens are the natural hosts for all viruses of the leukosis/sarcoma group. However, other natural hosts may also exist, but chickens are the species studied the most due to the consequences of ALV-infection in chicken production. Experimentally, some ALV subgroups have a wide host range and can be adapted to infect unusual hosts by passage in very young animals. ALV-J seems to have the widest host range, probably owing to its receptor usage (see below). There are many studies of seroprevalence and/or the presence of ALV-A, ALV-B and mainly ALV-J in several wild and domestic bird species other than chickens, which are not necessarily related to clinical disease. A better estimation of the host range of ALV, especially of the emerging ALV-J, is important to better understand the role that other birds may play in ALV epidemiology, transmission to other birds and spread to new regions. For example, the presence of ALV-J in Anseriformes and other migratory birds highlights the potential role of wild-bird migration in the spread of ALV-J worldwide [117].

The incidence and relevance of the infection is different in birds in general and chickens specifically raised in commercial and non-commercial flocks.

### 4.2. ALV Prevalence in Broilers and Layer Poultry (Commercial Poultry)

Infection by ALV-A/B/C/D has been practically eliminated from breeding flocks of most international breeding companies (broilers and layers producers), mainly in major developed countries and regions like the United States and Europe, due to strict eradication programs and management measures (reviewed in [20]). In addition, several countries, including China, Australia and Iran, have developed their own eradication programs (reviewed in [55]). However, the spread of ALV remains a major concern worldwide, and is even emerging in different areas, such as in China. The emergence of ALV-J in China may be also related to the difficulty in implementing eradication programs, the carrying out of which is problematic on small-scale farms (yellow-chicken local breeds) because of the lack of financial or technical support [21,22].

ALV-J was first isolated from broiler breeder chickens in the UK in 1988 and in the early 90s in the United States and Japan. It has rapidly spread around the world, causing severe economic losses to the poultry industry in several countries and regions such as Europe, East Asia (China, Malaysia, Taiwan), Russia, Australia, Israel and Egypt [22,47,118,119]. In recent years, the host range of ALV-J infection has expanded [47,120] from broilers to laying hens, local native chicken breeds [4,19,118] and gamecocks [22]. Its geographical spread, increased pathogenicity and faster transmission ability explain why it is the most prevalent subgroup nowadays [1,47,50,121]. For example, in China, one of the countries most affected by ALV, the most prevalent nowadays is ALV-J, followed by subgroups ALV-A and ALV-B, while subgroups ALV-C and ALV-D are seldom reported [21]. A survey in Russia showed that 70% of 223 production sites from 46 regions were seropositive to ALV-J, and that 90% were seropositive for generic ALV [55].

ALV-K was first isolated from a Chinese native indigenous chicken breed, “Luhua”, in 2012 [21,58]. Shortly afterward, several ALV-K strains from other yellow-feather broilers and Chinese indigenous or native chicken breeds in South China were described [43,60,122]. It is believed that ALV-K has existed in the indigenous chicken breeds of East Asia for a long time. Subsequent epidemiological investigation revealed that ALV-K was widespread among Chinese native chickens [43]. Most ALV-K were isolated from apparently healthy individuals, but some variants are significantly pathogenic [55,59,60,123,124].

### 4.3. ALV Prevalence in Backyard, Fancy and Hobby Chickens (Non-Commercial Poultry)

The rearing of small poultry flocks in backyards for eggs and meat, even sometimes kept as ‘pets’ in private gardens in cities, has gained popularity in western countries. These backyard poultry flocks consist mostly of native layer hen breeds, but also include fancy chickens and other domestic gallinaceous birds, such as turkeys, quails and geese, that are often obtained from various sources. The biosecurity measures of these non-commercial flocks are much lower than in bigger poultry farms, and they potentially may represent a risk in the transmission of viruses to other wild or domestic birds [20,125,126,127] and there is little knowledge of the diseases that these chickens could be at risk of contracting. In addition, strategic programs for the control of ALV infections have not yet been implemented in these type of flocks [20].

Data on the presence of ALV in backyard or in fancy chickens are very scarce and are often derived from questionnaires and post-mortem examinations (such as the myxosarcoma associated with ALV-A infection reported in a flock of mixed fancy breed chickens in USA in 2010 [128]), so its prevalence could be underestimated. ALV-induced leukosis/sarcoma diseases were presumptively diagnosed in 3–4% of birds in backyard poultries in USA [129,130], but no suspected cases were reported in a study in Finland [126]. 

One study on pure-fancy chickens in Germany using ELISA detected the presence of p27 (CA) in cloacal swabs in 28.7% of individuals and 56.0% of the 50 flocks tested [20], higher than the prevalence of antibodies previously reported in Switzerland in 2002 (2–4%) [131] and in the Netherlands in 2004 [132]. ALV-K was the subgroup identified in fancy chickens in Germany which also differs from the detection of ALV-A, B, and J identified in the Netherlands [132]. Therefore, the high proportion of fecal shedders suggests that fancy chickens should be considered a potential reservoir for ALV. High priority should be given to biosecurity by poultry farmers and veterinarians to prevent the introduction of the virus on commercial poultry farms, especially because of the recent increase in free-range management in commercial egg and poultry meat production [131].

In 2018, an ALV-J associated myelocytoma was described in a hobby chicken in the UK, a 12-month-old Black Rock chicken from a mixed breed flock of 6 birds. This chicken had respiratory and paralysis signs, and tumor lesions were observed in several organs that tested positive for p27 (CA) by ELISA and positive to ALV-J by PCR [127]. This was the first time that ALV-J had been detected in the UK since its eradication in the 1990s, which suggests that ALV-J may be circulating at a low level in backyard chickens, thus representing a possible reservoir of infection for other birds. 

Besides domestic fowl, host-range studies showed that red junglefowl (*Gallus gallus*) and Sonnerat’s junglefowl (*Gallus sonneratti*) are susceptible to infection by ALV-J (HPRS-103). These findings highlight the importance of continued surveillance of backyard, fancy and hobby chickens to detect potential new and re-emerging disease threats, such as ALV-J and ALV-K, which may be of significance to the wider poultry population [127].

### 4.4. ALV Prevalence in Non-Chicken Species 

There have been many studies looking for potential new hosts for ALV in different types of birds: domestic (turkeys, quails), urban (pigeons) and different captive and wild bird species, including passerines, columbids, waterfowl, and psittacines [125]. While ring-necked pheasant, Japanese green pheasant, golden pheasant, Japanese quail, guinea-fowl, Peking duck, Muscovy duck and goose are considered to be resistant to exogenous ALV [133], it has been known for some time that RSV can cause tumors in pigeons, pheasants, ducks, turkeys, rock partridges (*Alectoris graeca*) depending on the RSV subgroup/pseudotype. There are also several studies that demonstrate the susceptibility to ALV infection in vitro of cells derived from different species of birds, such as quail cells [134], related to the presence of a compatible receptor (Section 5), making it feasible that ALVs infect these species. This has brought concerns of transspecies ALV transmission, mainly ALV-J due to its higher transmissibility and broader range of hosts. The transmission to new species could establish natural reservoirs of circulating ALVs, not subjected to surveillance and control, and what is crucial, with a capability for further evolution [135]. This may represent also a challenge for the ecological balance [136]. The presence of the virus has been detected in different captive and wildlife birds in numerous countries, mainly in China and East Asia. This a priori means that these birds would be susceptible to infection in vivo, and perhaps play an epidemiological role in their transmission, carrying and spreading the virus to other birds. 

Turkeys are important domestic bird species that are reared on a large scale in many countries. Turkey cells are susceptible to ALV exogenous subgroups, although some show resistance to ALV-B, D, and J [121,137]. In 2017, Zeghdoudi et al. [138] reported an outbreak of tumor disease in turkey flocks in Eastern Algeria, with a mortality rate of 10% in animals aged 17 weeks and older. Tumors tested positive to the ALV p27 (CA) antigen by ELISA. Venugopal et al. [137] showed that 1-day-old turkey poults are experimentally susceptible to infection with ALV-J strain 996, a *myc*-expressing, acutely-transforming recombinant, that induces tumors with histopathological lesions of myelocytomatosis between 3 and 4 weeks after infection. In the same study, the authors also reported that ALV-J could spread among turkeys by contact, although at low levels, which stresses the importance of segregating turkey- and chicken-breeding operations to avoid the spread of ALV-J infection. This would be especially relevant in familiar small farms and backyards. 

Quails are very popular gamebirds that are raised in game farms for hunting purposes or other uses together with other gamebird species such as pheasants and partridges. ALV-A has been detected in quails in China [139], and there are several reports on the ALV receptors and the susceptibility of quails to ALV-A and ALV-J experimental infections [135,136,140]. Zhang et al. [136] reported that adult quails were less susceptible than chickens to experimental infection by ALV-A because only a few infected birds showed transient viraemia, low cloacal virus shedding and antibody response, which might limit their role in bird–to–bird transmission. New world quails are known to be susceptible to ALV-J infection because they have a receptor compatible with virus entry [135]. 

Partridges, closely related to pheasants and grouse, have been previously described as ALV-J resistant [133]. However, Shen et al. [141] concluded that the grey partridge (*Perdix perdix*) may develop ALV-J-linked tumors when bred together with infected susceptible species, such as black-bone silky fowl (*Gallus gallus domesticus* Brisson), effectively showing that housing different avian species together provides more opportunities for ALV-J to evolve rapidly and expand its host range [141]. 

The presence of ALV-A, ALV-B, and/or ALV-J has been reported in wild ducks from the order Anseriformes and some species of small Passeriformes in China [117,142,143]. ALV-J has been detected in wild ducks, though Zeng et al. [144] could not determine whether ducks are susceptible to ALV-J infection or simply carriers of it following contact with domestic poultry harboring very high loads of virus, as they could not perform serology. However, it would not be surprising to find that wild ducks are truly susceptible to ALV-J, as poultry and wild ducks are evolutionarily more closely related than poultry and passerine birds, and wild ducks tend to carry and spread poultry pathogens easily [143]. Later findings by Reinišová et al. [145] confirmed that certain Asian ALV-J strains had coevolved and gained the capability to enter duck cells. Surprisingly, the sequencing of ALV-J strains from passerines showed their similarity in the 3’UTR to those found in Chinese layer chicken isolates, indicating that both may have a common origin, which points to the possibility of a convergent relationship that needs further investigation [143].

Pigeons are reared in many countries, as well as being very common urban birds in cities worldwide. To the best of our knowledge, there is only one study by Zhang et al. [136] describing the absence of viremia, cloacal virus shedding, antibody responses, clinical signs or pathological lesions in experimentally ALV-A-infected adult pigeons. These results suggest that pigeons are resistant to ALV-A infection, although the susceptibility to ALV-J has not been studied.

Natural infection by ALV-A through E has been reported in other birds, including two green peafowl (*Pavo muticus*) with neoplasia [146] and in wild ostriches from a farm in Zimbabwe, which tested positive for antibodies and at least one case had clinical illness [147].

All these facts raise questions about the presence of ALV in wild birds other than chickens and close relatives, but few such studies exist. In conclusion, the ALV host range is broader than just the Galliformes; but further research is needed to determine which avian species are susceptible.

### 4.5. Endogenous ALVs in Non-Chicken Species

Non-chicken species may be infected both by ALV-E and by other endogenous retroviruses. Host range experiments show that although jungle fowl, Peking duck and goose cells are resistant to ALV-E, this subgroup may infect pheasant, Japanese quail, Guinea fowl and turkey cells in vitro [5].

ALV and related viruses have infected the germinal line of Galliformes at several points in time [148]. Subgroups F, G, H and I, sometimes described as endogenous ALVs, were also considered ALV but research at the time [149] found big differences between them and the canonical ALVs in hybridization studies. Since then, few research efforts have been made on subgroups F to I and a lack of a complete model sequence in databases prevents further advance, as mentioned above. Nonetheless, they are still considered ALVs.

ALV-F was first described in ring-necked pheasants (*Phasianus colchicus*) in 1973 [150]. A year later, ALV-G was found in golden pheasants (*Chrysolophus pictus*) and was recognized as different from ALV-F [151]. ALV-H and ALV-I were found in Hungarian partridge (*Perdix perdix*) and Gambel’s quail (*Callipepla gambelli*) endogenous sequences, respectively [152]. Dimcheff et al. [153] reported that *gag* regions similar to those of ALV exist in the genome of a wide range of 26 different Galliformes species such as grey partridge (*Perdix perdix*), black grouse (*Lyrurus tetrix*) or helmeted guineafowl (*Numida meleagris*) and even drafted the complete sequence of TERV (Tetraonine endogenous retrovirus), an ASLV from the ruffed grouse, *Bonasa umbelus* [154] but could not determine whether they were of exogenous or endogenous nature, due to the high conservation of *gag* sequences. Though Zhu et al. described the detection of the ALV *gag* gene in the genomic DNA of eight Chinese native domestic geese (*Anser anser domesticus*) [155], these results were later refuted by Elleder and Hejnar, as it is possible that PCR amplifications were contaminated with chicken genomic DNA [156]. According to Dimcheff et al. [154], endogenous ALV is restricted to Galliformes and is not present in the closely related Anseriformes order or the families *Cracidae* and *Megapodiidae*. However, later research by Hao et al. [157] found proviral ALV-E in wild duck samples [155], increasing the range of species where ALV proviruses are present (Table 1). We have found ALV *env* sequences in genomic scaffolds from the *Alectoris rufa* genome that was recently published (UCSC genome browser Id: GCA_947331505.1; unpublished results) and also in *Tympanuchus* and *Lagopus* (both of the family *Phasianidae*) published RNA sequences (GenBank Accession Numbers XM052699038 and XM042864983, respectively). The presence of endogenous ALV in *Tympanuchus* and *Lagopus* was also reported by Dimcheff et al. [153] and for a different species of *Alectoris* by Frisby et al. in 1979 [158]. 

## 5. Different ALV Subgroups Use Different Cell Receptors

**Figure 6:**
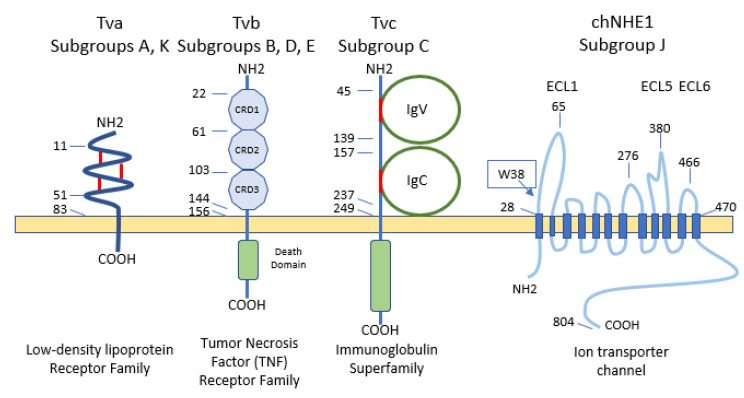
Schematic representations and residue position of critical domains of the four families of cellular surface proteins which used ALV receptors (adapted from [39,167]). CRD, cysteine-rich domains. IgV, immunoglobulin variable domain. IgC, immunoglobulin constant domain. ECL, extracellular loop. The position of tryptophan-38 (W38) is shown in the ECL1 of chNHE1.

The in vitro tropism of ALVs is quite flexible and the infection has been reported in a variety of cultured cells from different avian species. For in vitro culture, primary chicken embryo fibroblasts (CEF) are the most common cell type used, and some CEF phenotypes such as C/0 are sensitive to ALV-E infection [5,159]. DF-1 cells are also commonly used. These cells, while free of endogenous ALV, are C/E as they do not have an ALV-E-susceptible receptor phenotype (they are derived from Line 0) [160]. ALV enters susceptible cell types by binding to specific receptors [21,33] (Figure 6). The analysis of the different susceptibilities of various chicken cell lines to infection by ALV has been useful for identifying three distinct autosomal recessive genes, which were considered to encode the cell receptors for the different ALV subgroups, and helped to describe the concept of viral interference [161].

ALV-A enters the cell through the Tva protein, which is homologous to a low-density lipoprotein receptor in humans [5,21,33]. However, ALV-A may broaden its receptor usage to other receptors in the presence of a competitor of Tva, SUA-rIgG immunoadhesin [162]. Tva is also the receptor for ALV-K [163]. The viral interaction domain of Tva is determined by a 40-aa-long motif which interacts with key amino acid sites, modulating the affinity, and variations in these aa result in a weaker replication ability of ALV-K than ALV-A [59,142,164].

The gp85 (SU) glycoproteins of ALV-B/D/E bind to the three extracellular cysteine-rich domains (CRD) of Tvb receptors, which belong to the tumor necrosis factor receptor family (TNFR). Mutations in these domains modulate the susceptibility to the mentioned ALV subgroups [21] and it has been proposed to generate resistance to ALV [165]. As in other retroviruses, modifications in the sequence of gp85 (SU) also result in decreased susceptibility to infection by these three subgroups. The normal cellular function of chicken Tvb remains unclear, though it may be similar to the other members of the TNFR family. Besides the CRDs, Tvb also has a cytoplasmic death domain that can activate cell apoptosis through the caspase pathway and produce a cytopathic effect [5,116].

ALV-C binds to Tvc, a member of the butyrophilin family of the immunoglobulin superfamily. The Tvc extracellular tail contains two immunoglobulin globular domains, IgV and IgC. The gp85 (SU) of ALV-C binds to IgV which has two aromatic aa residues (Trp-48 and Tyr-105) which are key determinants of receptor–virus interactions. In addition, a domain such as IgC seems to be necessary as a spacer between the IgV domain and the transmembrane domain for efficient Tvc receptor activity, most likely to orient the IgV domain a proper distance from the cell membrane [166].

Subgroup ALV-J uses the 12-span transmembrane Na^+^/H^+^ channel protein type 1 (chNHE1) as a cell receptor [5,33,167], sometimes referred to as Tvj. NHE1 is a housekeeping protein that regulates intracellular pH, Na^+^ and H^+^ ion transport and cell proliferation; it is expressed on almost all cell membranes [168] and chicken breeds, possibly rendering all chickens susceptible to ALV-J [48]. It has been reported that the expression of mRNA and protein of *NHE1* can be induced by ALV-J, and chickens infected by ALV-J develop a more complex array of clinical signs, probably due to the relevance of the receptor in cell biology. The consequences of the increased levels of NHE1 may be multiple as it is involved in the intracellular pH balance, apoptosis and cell proliferation, all of which are seen to be altered in ALV-J infection. This may explain why ALV-J infection leads to a high development of tumors in different organs (bone marrow, liver and kidneys) [21]. It has been reported that the deletion or substitution of tryptophan in position 38 (W38) increases resistance to ALV-J [5,169] (see Section 7.3.1). Besides chNHE1, chicken annexin A2 (chANXA2) [170] and the chicken glucose-regulation protein 78 (chGRP78) [21] have been described recently as receptors for ALV-J, possibly as secondary receptors in the absence of chNHE1.

## 6. Immune Response against ALV Infection

Immune responses to ALV are complex and not fully understood, and many questions remain unsolved. In addition, ALV, specially ALV-J, are well known immunosuppressive viruses and can suppress innate and adaptive immune responses [33,171], affecting the monocyte, macrophages, dendritic cells (DC), B- and T-cell functions. In recent years much more attention has been paid to the innate immune responses and how ALV can evade them.

### 6.1. Innate Immunity

Host immune response against ALV is initiated with the recognition of the virus by the innate cells. It was reported that the viral RNA of ALV could be recognized by TLR7 and MDA5 (melanoma differentiation–association gene 5, a member of the RIG-I-like helicase receptors (RLRs) family), inducing the expression of interferon-stimulated genes (ISGs) and inflammatory cytokines [172]. However, data of other innate sensors and mechanisms of cellular innate immunity are quite limited [173,174].

A well-known mechanism of the cellular innate defense against retroviruses in mammals is the Host Restriction Factors (RFs), antiviral molecules that inhibit the retroviral replication cycle at multiple steps, and that are often up-regulated by type I interferons. Few RFs have been identified in chickens that are able to restrict ALV replication: the cellular scaffolding protein Daxx promotes the epigenetic silencing of integrated proviruses [175]; CCCH-type zinc finger antiviral protein (ZAP) inhibits ALV-J replication by interaction with mRNA and SU protein [176]; TRIM62 restricts ALV-J replication through the SPRY structural domain [177]; TRIM25 inhibits ALV-A replication by regulating MDA5 [178]; cholesterol 25-hydroxylase (CH25H) probably inhibits viral entry through a not completely understood mechanism [179]; and a recently identified tetherin/BST-2 protein blocks the release of newly formed viral particles by linking them to the membrane of infected cells [180]. One of the most widely studied RFs, apolipoprotein B mRNA-editing enzyme-catalytic polypeptide-like 3 (APOBEC3), is not present in birds. A better knowledge of the avian RFs could be also very interesting because they are key determinants of cross-species transmission, as has been demonstrated in mammal retroviruses. In addition, understanding the mechanisms of RFs interaction with ALV may be used to develop novel antiviral strategies in poultry [176].

The role that non-coding RNAs play in the host innate response to ALV has received much attention in recent years. Host microRNAs (miRNAs) interfere with cell growth, proliferation and apoptosis and their aberrant expression may be associated with neoplastic diseases; but they are also involved in the regulation of the host innate response. Several miRNAs, such as miR-34b-5p and miR-23b, were significantly increased in the spleen of ALV-J-infected chickens (reviewed in [174]). miR-34b-5p inhibited the expression and activation of MDA5, facilitating ALV-J replication [181]. miR-23b could decrease the expression of interferon regulatory factor (IRF)-1 and further down-regulate the expression of β-interferon (IFN-β), which facilitated ALV-J replication [182]. On the contrary, in ALV-J infected cells the expression of miR-125b was down-regulated and apoptosis inhibited [183]. These data suggest that ALV could regulate in some way the expression of host miRNAs to target host proteins, inhibiting host anti-viral response. However, more efforts are needed to elucidate this mechanism of innate immune evasion by ALV [174]. 

Monocytes, macrophages and DCs are the pivotal innate cells against viral infection and there are several studies about the effect of ALV infection, mainly ALV-J, on this cell lineage. ALV-J infection of monocytes induces cell death, but the underlying mechanism is still unclear although there is an increase in the activities of caspase 1 and 3 and up-regulation of IL-1β and IL-18. Therefore, infected monocytes cannot differentiate to macrophages, altering the innate and adaptative immune response [184]. Previous studies by Gazzolo et al. [185] showed that ALV-B/C (but not ALV-A/D) viruses could persist in macrophages from peripheral blood up to 3 years, in 1- or 2-month-old chickens immunized against these viruses. The monocyte-derived macrophages (MDM) infected with ALV-J secrete more IFN-β and pro-inflammatory IL-1 and IL-6, and less anti-inflammatory IL-10, which might suggest that this is a strategy for ALV-J to evade host innate immunity and even establish latent infections in macrophages and serve as cellular reservoirs [173]. In addition, IL-1 and IL-6 have been linked to tumorigenesis, which suggests that inflammation would be associated with tumor development [186]. Other studies described the inhibition of type I IFN, TNF-α, IL-1β, IL-6 and IL-12 production in infected ALV chicken macrophages. These discrepancies observed in different studies may be related to the use of macrophage cell lines, instead of MDM, in which immune functions can be impaired and genetic differences may have developed with continuous subculture [186]. As in monocytes, ALV-J are able to infect chicken DCs and disturb their normal functions, including their rate of maturation, while inducing apoptosis and causing aberrant expression of microRNAs [187]. ALV-J also decreases the expression of TLR1, 2, and 3 and of the major histocompatibility complex (MHC) classes I and II [188], and alters cytokine expression. All this causes aberrant antigen presentation and an altered immune response. These alterations in DC and monocyte/macrophage functions may be involved in the immunosuppression of the host innate immune response during ALV-J infection [187].

What are the subcellular mechanisms underlying ALV interference with the host innate immune response? Although they are not fully known, it appears that the virus targets host proteins involved in signaling pathways of the innate immune response. Some studies have reported that ALV infection can, among others, (a) up-regulate the expression of SOCS3, thus inhibiting the phosphorylation of the JAK2/STAT3 pathway [189], (b) block phosphorylation of IκB, avoiding the dissociation of the complex with NF-κB, which does not translocate into the nucleus to activate immunoregulatory genes [190], and (c) up-regulate the disruptor of telomeric silencing 1-like protein (DOT1L), inhibiting the expression of MDA5 (which is a cytosolic double-strand RNA sensor), thus affecting the recognition of ALV RNA by host pattern recognition receptors (PRRs) [191]. As a result in all cases, the activation of the type I interferon signal transduction pathway is impaired [174,178]. On the other hand, miRNAs and other small RNA molecules (piRNA and lncRNA) and proteins derived from endogenous ALV can sense or modulate immune responses against viruses [21]. As mentioned above, despite the evidence for innate immunosuppression caused by ALV, there are still many unresolved questions to understand the underlying mechanisms by which ALV inhibits the host’s immune response. 

### 6.2. Humoral Immune Response: The Role of Antibodies

The humoral immune response to ALV mainly depends on the timing of infection (congenital vs. horizontal), the type of viral subgroup involved and the genetic basis of resistance of the chicken. After horizontal transmission, most of the chickens infected by ALV subgroups A and B (unlike those infected by ALV-J) develop a transient viremia within a week, followed by neutralizing antibodies at three weeks or later directed against the envelope proteins that rise to a high titer and have a lifelong persistence, preventing the reappearance of viremia [192]. These neutralizing antibodies reduce the amount of virus in the body, which in turn will limit tumorigenesis, but they generally are considered to have no or an undetectable direct influence on tumor growth [193]. In addition, neutralizing antibodies enhance virus evolution in order to escape their blockage. The fact that in ALV-J-infected chickens levels of viremia do not correlate with the presence or absence of neutralizing antibodies is associated with the development of escape variants [194,195]. The presence of maternal antibodies against ALV-A in chickens hampers the development of neutralizing antibodies by the chick, allowing viremia and virus shedding [192].

Congenital and early infections (<2 weeks of age) cause immunological tolerance with no humoral response to the virus, and infected chickens develop a persistent viremia in the absence of neutralizing antibodies [196,197]. In particular, ALV-J induces a severe and persistent immunotolerance in congenital infection through mechanisms which are not completely elucidated. This immunotolerance is characterized by persistent viremia, dysplasia of lymphoid organs and severe decrease in the ratio of CD4^+^/CD8^+^ T-cells in blood and lymphoid organs, CD3^+^ T-cells and B-cells in the spleen [198]. The bursa of Fabricius is poorly developed and its structure does not differentiate into cortex and medulla (this is not the case in post-hatch infection, in which the bursa development is relatively normal [199]) due to the blocking of the differentiation of B-cell progenitors, causing an arrest of the development of B-cells and the inhibition of humoral immunity; in addition, ALV-J gp85 (SU) mediates B-cell anergy by inhibiting BCR signal transduction [199]. Congenital ALV-J infection also induces the production of activated CD4^+^CD25^+^ Tregs that inhibit B-cell functions maintaining the persistent immunotolerance [198].

Depending on the humoral immune response and the presence of a detectable virus in the blood, susceptible and infected ALV chickens can be classified in several conventional categories: viremia with (V+A+) or without (V+A−) neutralizing antibodies, and no viremia with neutralizing antibodies (V−A+) [15,200,201]. Most of the infected chickens are V−A+. Less than 10% are V+A− (immunotolerant chicken, infected congenitally or at a very young age) and transmit the virus to other birds and to their progeny, and are likely to develop lymphoid or myeloid leukosis (reviewed in [1,5]); this may not be the case in ALV-J infected chicken. V+A+ are usually those in the process of clearing an acute infection, eventually becoming V−A+ [15]. However, the plasticity of ALV-J and its capability of generating escape mutants is readily appreciated through sequencing the consecutive ALV-J isolates from V+A+ chickens, which show evolution, thereby contributing to viral persistence [195]. Genetically resistant birds in a susceptible flock and those in an infection-free flock are V−A−.

### 6.3. Cellular Immune Response: The Role of T-Cell Dependent Cytotoxicity

Initial studies showed that cytotoxicity by T-cells seemed to play a role in the susceptibility or resistance of the various MHC-I haplotype chicken lines to ALV-A infection [202]. A role for cellular immunity was also correlated with immunosuppression of the T-cell function in ALV-A infected chickens [203]. 

Once again, most of the evidence for the involvement of the cellular immune response in ALV infections comes from studies with ALV-J. In experimental infections with ALV-J, CD8+ T-cell response was detectable by 7 days post infection (dpi), while specific antibodies were detected from 14–21 dpi in some chickens. As viremia levels decrease from 14 dpi, and are generally negligible by 21 dpi, it is evident that humoral response is not solely responsible for viral clearance [204]. Dai et al. [204] also reported that viremia of four experimentally infected ALV-J chickens could be eliminated without antibody production, which suggests that CD8^+^ T-cell response was the potential key factor to defend against ALV-J infection. This was supported by the detection of an increased percentage of CD8^+^ T-cells of phenotype CD8αβ (CTLs) in the thymus and peripheral blood lymphocytes (PBL) at 7 and 21 dpi, and an up-regulated expression of two antiviral ISGs (Mx1 and IFIT5) and the cytotoxicity-associated genes granzyme K, NK lysin and IFN-γ, linked with the stage of activation of the CTLs and the clearing of viruses. However, Dai et al. [204] also detected a decrease in the CD4^+^/CD8^+^ ratio in the thymus at 14 dpi and in the PBL at 21 dpi, which implied that the virus may have exerted an immunosuppressive effect at this period but also that CD4^+^ T-cells could be a primary target for ALV-J [173]. 

It has been previously observed that infection by ALV-J produces an inhibition of blood and splenic T-cell proliferation and cytotoxicity in broilers [205] and a reduction in CD4^+^ and an increase in CD8^+^ T-cell populations in the spleen [206]. Taking all the data together, it can be concluded that ALV-J exerts an immunosuppressive effect on both humoral and cellular responses and that 3–4 weeks post-infection may be the critical period for this ALV-J inducing immunosuppression. 

All these results help to better understand the cellular immune response against ALV, although further studies are needed to elucidate the specific roles of T-cells against ALV during natural or experimental infections. A better knowledge and understanding of humoral and cellular immune responses may help to develop breeds with increased resistance to ALV and more effective vaccines.

## 7. What Can Be Done to Control ALV Infections?

### 7.1. Prevention through Vaccines

Many vaccine candidates have been tested with limited success, and currently no commercial vaccine or effective treatment against ALVs exist [33]. Different approaches have been used to design vaccines, and though the production of attenuated live ALV has failed [5], other strategies seem to be more promising. These include recombinant vaccines with ALV-J *env* or the part encoding gp85 (SU) combined with adjuvants [207]. New adjuvants include immunomodulators such as CpG-ODNs, as agonists of chicken TLR21, that in combination with a gp85 (SU) recombinant protein induce higher titers of serum antibodies in vaccinated hens and a better protection of the offspring through the maternal antibodies against ALV-J and ALV-A early infections [208]. Despite the promising results obtained in experimental studies, vaccines against ALV are not effective in the field for several reasons: difficulties in the administration route; the two routes of virus transmission, vertical and horizontal; the ability of ALV to mutate or recombine into new retrovirus strains; and that congenitally infected chickens will not react against the vaccine components as they are immunologically tolerant [5]. Nevertheless, a vaccine trial using *Lactobacillus plantarum* NC8 strain engineered to express ALV-J gp85 (SU) protein has been recently reported. This live vaccine can be orally administered, which solves the most limiting aspect of ALV vaccine development which is the cost of inoculating a flock. The NC8 strain expresses fibronectin-binding protein from *Staphylococcus aureus* which acts as an immune modulator and has been used successfully in vaccine research against several viruses in recent years [209].

As regards treatments, to date no drugs have been described that can be applied or are effective in the field. Many studies focus on the interaction between ALV and host factors to find molecular targets to block viral replication. In vitro approaches show that recombinant chicken IFN-α [210,211], zidovudine and short hairpin RNA targeting the reverse transcriptase of ALV and other interference RNA (RNAi) inhibit ALV replication in culture [212]. Though unlikely in field conditions, these in vitro experiments have shown a reduced viral replication and shedding after treatment and thus may be used in cellular cultures for vaccine production. On the other hand, neoplastic complications of ALV-induced disease cannot be treated with reproducibility in infected chicken. 

### 7.2. Selection of ALV-Free Poultry

Since immunization or treatments are not currently commercially available, the current strategy for ALV control is eradication of the infection from breeding stocks. Poultry producers analyze their birds in order to eradicate ALV in a breeding lineage by discarding congenitally infected individuals until part of the flock can be considered ALV-free and serve as replacement stock [1]. The recommended samples and tests for selecting dams free of ALV are vaginal and cloacal swabs and egg albumen for the p27 (CA) antigen. Detection can be achieved by ELISA, though endogenous p27 (CA) antigen may interfere with the test, and it is necessary to discriminate between actual virus shedding and ALV-E presence. It is advisable to perform several tests since only one may not be enough to identify all chickens shedding the virus [5]. Positive dams are removed because they are likely to transmit the virus to progeny chicks. In addition, chickens from the next generation need to be screened by PCR or other tests for the presence of the virus, and individuals that test positive and their contact birds are discarded. In practice, it is easier to select hens free of ALV than chicks testing and rearing them in isolation and commercial breeders concentrate only on hen analysis [5]. Hatching of chicks in isolation in small groups in wire floored cages and with adequate biosecurity measures are also useful to reduce the horizontal transmission of ALV [213].

### 7.3. Resistance to ALV through Manipulation of Bird Genomes

Chickens naturally display some resistance to ALV infection. In the 1940s, chickens were selected for higher broiler and egg production; however, breeders unknowingly selected phenotypes that were susceptible to ALV infection. Nowadays, most commercial flocks have been subjected to selection to improve their resistance to pathogens [89]. The resistance mechanisms are engrained at different levels: cell receptor, IFN genes, ALV-E provirus inducing ERV-derived immunity, miRNAs or others. All these form multiple protective layers for chickens and work coordinately to avoid infection or ease clinical signs such as oncogenesis. Nearly all these barriers have resistant and susceptible alleles or can be engineered to improve chicken immunity and response to ALV.

#### 7.3.1. Boosting of Host Resistance by Cell Receptor Genetic Edition

One of the most studied mechanisms of resistance is the role played by editing the cell receptors that mediate viral entry in the cell, which are highly polymorphic between bird species and within chicken lineages. For the A to E subgroups, cellular resistance depends on *tva*, *tvb* and *tvc* genes that encode the cell receptor for viral entry in the different subgroups, with sensitive (s) or resistant alleles (r) [5]. A *tvb* allele, *tvb^T^*, identified in turkeys, has been demonstrated to confer susceptibility to ALV-E infection, which acts as an exogenous virus in this species [33,214]. *Tva-* and *tvb*-resistant alleles may be found enriched in Chinese local breeds, making them desirable for selection or gene-editing strategies that will be explained below [21,215].

Tryptophan in position 38 (W38, corresponding to the extracellular loop 1, ECL1, Figure 6) of chNHE1 mediates the resistance/susceptibility to ALV-J [169]. Both its substitution (as in Japanese quail) [5] and its deletion (as in the grey partridge or in Anseriformes) render the bird resistant to ALV-J. In addition, the introduction of W38 to NHE1 devoid of it confers susceptibility to previously resistant host species [135]. New World quails are susceptible to ALV-J infection because they have a functional receptor which contains a tryptophan residue homologous to W38 of chNHE1 [135] and in Anseriformes it may be a different motif [145,216]. 

Assays editing chNHE1 have recently been performed and proved that W38 deletion can be engineered, granting resistance to ALV-J infection [5,217]. Edition of the cell receptor *tvb* has been tested in DF-1 cells and their resistance to infections by ALV subgroups that use this receptor was found to be increased [165]. CRISPR/Cas9 may be used to edit the receptor genes. Koslova et al. [218] used this technology to introduce frameshift mutations in the genes *tva*, *tvc* and *chNHE1* to generate early stop codons, and demonstrated that these cells became resistant to the subgroup whose receptor was edited. The same principle may be used for generating new resistant chickens. Chickens resistant to ALV-A and ALV-K can be obtained by knockout of *tva* gene with CRISPR/Cas9 since it is known to not be necessary for healthy chicken growth and other signaling molecules may compensate for the loss of Tva [21,217]. 

#### 7.3.2. Other Determinants as Targets for ALV Resistance

Resistance to ALV infection is polygenic; thus, another strategy is increasing the frequency of resistant alleles to ALV infection in a flock. Genotypes may be determined by mating individuals with chickens that carry double recessive alleles for the subgroup of interest and testing the progeny [5,21]. 

Other genes that determine resistance to ALV are those that hamper tumor progression. For example, it is known that some MHC-I haplotypes, such as B12, confer resistance to RSV by enabling binding to >10 times more peptides [219], and something similar could occur in ALV infection. Genes related to interferon synthesis, such as ISGs, are also relevant in ALV replication; some of them have antiviral properties, including ACSL1 which up-regulates IFN-I expression, promoting apoptosis through PI3K/Akt pathways, effectively inhibiting ALV-J replication [220]. Mountford et al. ([221]) found 60 variants of IFN pathway genes in chickens that may account for resistance vs. susceptibility to viral pathogens. However, further research is needed to understand the acting mechanisms of the hundreds of ISGs known to date [21]. Once these mechanisms are better known, they may be targets for selection or edition.

Some micro RNAs (miRNAs) are also involved in ALV pathology, such as miR-23b, which down-regulates IRF-1 and promotes ALV-J infection [182] (read above). These miRNAs are repressed by the action of circRNAs (non-coding circular RNA), such as circ-vav3 which is the sponge of miRNA gga-miR-375 and inhibits its function in ALV-J infection. This interaction up-regulates YAP-1 (*yes*-associated protein 1) which in turn, promotes tumorigenesis [222]. Zhang et al. [223] have found a plethora of circRNA and miRNA to be up-regulated in breeds of chicken that are resistant or susceptible to ALV. These microRNA may be desirable selection or edition targets against ALV tumorigenesis and circRNAs may be used as molecular markers of ALV-J-resistance in chickens.

Other genes are induced upon ALV infection; some have antiviral effects while others may promote viral replication and may be used for selective breeding. Single Nucleotide Polymorphisms (SNPs) can be used to detect resistant genotypes and thus may also be used as a selection target [224]. Lastly, hormones have important effects over host immunity and viral replication [225,226], so determining the desired hormonal profile for chickens and directing selective breeding towards that goal is also a recommended practice [21]. Mo et al. [227] described how prolactin and growth hormone expression was higher in the serum of early feathering chickens. Late feathering chickens were found to have higher immune-related gene expression in spleen than early feathering ones, albeit presenting lower IgG and IgM in serum. Finally, they reported that higher prolactin secretion promoted ALV-J replication, and they speculated that receptors for prolactin and decidual prolactin (a duplicated prolactin receptor present in late feathering chickens, in linkage association with *ev21* [65,66]) may somehow act as ALV-J receptors; however, they also showed prolactin has ALV-J antiviral properties [226]. In a study with a limited number of yellow chickens, a different group of hormones, luteinizing hormone, follicle stimulating hormone and progesterone, were found to be correlated with intermittent viremia but not with persistent viremia. This means that the reproductive stage might be relevant for testing for ALV presence in chickens [225] similarly to other retroviruses [228,229].

#### 7.3.3. Role of ALV-E Provirus in ALV Resistance

ALV-E infection induces tolerance to exogenous ALV subgroups but it also interferes with exogenous infection via ERV-derived immunity using several mechanisms such as receptor interference, capsid interference or regulation of host loci with effector functions [5,15,38,173,230] (see above and Figure 2). Viral LTRs in the host’s genome may regulate host genes, including immune response genes [21].

Particularly in ALV-E, inhibitory action of at least two anti-sense long non-coding RNA (Inc-RNA) over exogenous ALV infection has been reported. lnc-LTR5B regulates cell membrane translocation of BiP (Binding Immunoglobulin Protein), which is used by ALV-J to exit the cell. lnc-ALVE1-AS1 activates the TLR3 pathway to trigger the antiviral innate response [21,78]. Theoretically, the cDNA sequences that these lncRNA originate from could be engineered for higher expression or duplicated in a cassette to a certain part of the genome to increase resistance against ALV.

Some ALV-Es, such as EV21 and EV6, have been shown to increase susceptibility to ALV infection as a result of induced tolerance due to the high levels of envelope protein produced by these ERVs [36,64]. Unlike these, some ERV insertions may have commercial value, so a valid strategy is elimination of the negative ERVs by selection in a commercial breed while keeping a check that chicken growth is not hampered as a result [21].

## 8. Conclusions

Throughout this review it has been pointed out several times that many of the natural isolates may be mapped molecularly as recombinants between endogenous and exogenous sequences. Moreover, endogenous sequences do not necessarily need to be ALV-E, as was shown in the composition of ALV-J, the most problematic ALV nowadays from the economic losses point of view. Recombination is a recognized characteristic of retroviruses and it has contributed, for example, to the emergence of new subgroups of feline leukemia viruses [231,232] or porcine endogenous retrovirus capable of infecting human cells [233]. The possibility of recombination, along with the high mutation rate of retroviruses, allow ALV to develop escape mutants, a circumstance that may be frequent and hampers the effective control of ALV. Especially pressing has been the development of ALV-J, which uses a different receptor from the other subgroups, increasing its cellular tropism and transmissibility.

Another circumstance that makes control of ALV difficult is the possible infection of backyard, fancy and hobby chickens, and indigenous or native chickens, as they escape the control measures of the big international companies which produce commercial poultry. Though the presence of exogenous ALV in wild birds is limited, the possibility exists of recombination between exogenous ALV from backyard chickens and endogenous sequences of wild birds, including some lesser-known ones like ALV-F/G/H/I, which might generate transmissible ALV to domestic poultry or vice versa. On the other hand, great progress has been made on developing resistant poultry breeds through genetic engineering. In conclusion, we have the technology to modify the transmissibility of ALVs but attention should be paid to birds which are outside the realms of commerce or veterinary control.

## Figures and Tables

**Table 1:** Characteristics of ALV subgroups identified in natural infections. The most studied subgroups are shown in bold. ^1^, EX, exogenous; EN, endogenous. ^2^, Recombinants of the subgroup found in natural infections.

Alv Subgroup	En/ex ^1^	Cytophaticity	Recombination ^2^	Cell Receptor	Hosts
ALV-A	EX	NO	ALV-J	Tva protein	Galliformes (*Gallus gallus*; *Meleagris gallopavo*; *Pavo muticus*) Anseriformes (*Sibirionetta formosa*; *Anas carolinensis*)
ALV-B	EX	YES	ALV-J ALV-E	Tvb protein	Galliformes (*Gallus gallus*; *Meleagris gallopavo*) Anseriformes (*Sibirionetta formosa*; *Anas carolinensis*)
ALV-C	EX	NO	ALV-J	Tvc protein	Galliformes (*Meleagris gallopavo*)
ALV-D	EX	YES		Tvb protein	Galliformes (*Meleagris gallopavo*)
ALV-E	EN	-	ALV-A ALV-B ALV-C ALV-J	Tvb protein	Galliformes (*Gallus gallus*; *G. sonneratii* (EAV-HP); *Meleagris gallopavo*; *Lophortyx gambelii*, *Pavo muticus*) Anseriformes (*Anas falcata*; *A. carolinensis*; *Sibirionetta formosa*)
ALV-F	EN				Galliformes (*Phasianus colchicus*; *P. versicolor*; *Chrysolophus pictus*)
ALV-G	EN				Galliformes (*Chrysolophus pictus*)
ALV-H	EX				Galliformes (*Perdix perdix*)
ALV-I	EX				Galliformes (*Callipepla gambelii*)
ALV-J	EX		ALV-A ALV-B ALV-C	chNHE1	Galliformes (*Gallus gallus*; *Meleagris gallopavo*; *Perdix perdix*) Anseriformes (*Anas acuta*; *A. poecilorhyncha*; *A. penelope*; *A. carolinensis*; *A. crecca*; *A. clypeata*; *A. formosa*; *Sibirionetta formosa*; *Mareca strepera*) Passeriformes (*Tarsiger cyanurus*; *Emberiza elegans*; *Phylloscopus inornatus*; *Poecile palustris*)
ALV-K	EX		ALV-E	Tva protein	Galliformes (Chinese indigenous chickens: *Gallus gallus*)

## Data Availability

Data can be obtained from the corresponding authors or through the Animal Viruses UCM Research Group available at: https://www.ucm.es/animalvirucm/.

## Supplementary Materials

The following supporting information can be downloaded at: https://www.mdpi.com/article/10.3390/ani13142358/s1, Table S1: Validated ALV-E insertions in different breeds of chickens [234,235,236,237,238].
