# Supplementary material for: Avian Leukosis: Will We Be Able to Get Rid of It?
Source: Animals (Basel). 2023 Jul 19;13(14):2358. doi: 10.3390/ani13142358 (PMC10376345; doi:10.3390/ani13142358)
Supplement: Supplementary file 1 [file animals-13-02358-s001.zip › animals-2423198-supplementary.pdf]

Table S1: Validated ALV-E insertions in different breeds of chickens. Only elements validated by locus-specific assays and whose location is known are included. To the best of our knowledge, this is all the data available as of April 2023. Several endogenous elements are validated by different authors and they give slightly different loci for them. Displayed loci correspond to the written reference. An empty gene field only means co-localization with a gene hasn't been reported, not that there isn't a gene in that locus.

Locus	Integrity	Chromosome	Position	Gene	Reference
ALVE6	pol-env-LTR	1	210601		[234]
New34		1	9305977		[80]
ALVEB5	Complete	1	10637460	LOC112532986 int3	[63]
New8		1	16567873		[80]
Cotw46		1	18197112		[80]
Cotw47		1	26564943		[80]
ALVE-JFevB	Complete	1	32724216	Inside a GGERV20 ERV	[234]
New12		1	36572573		[80]
ALVE2		1	36872528		[81]
ALVE_CAU011		1	39162579	LOC101749434 int2	[237]
Cotw48		1	43582613		[80]
ALVE1	Complete	1	65993542	SOX5 int1	[63]
ALVE_CAU012		1	65996184	SOX5 int3	[237]
New9		1	66084636		[80]
New14		1	66142874		[80]
New31		1	66204837		[80]
Cotw53		1	66219813		[80]
ALVE16	LTR	1	67547189	In pol of CR1 element	[80]
New13		1	93369555		[80]
Cotw55		1	101007641		[80]

ALVE_ros001	Complete	1	101668931		[63]
ALVE_CAU016		1	102187653	NCAM2 int1	[237]
New10		1	110021612		[80]
Cotw57		1	111805576		[80]
New33		1	113591212		[80]
ALVE12	Partial deletion in 3' LTR	1	122259940		[81]
Cotw67		1	123254017		[80]
New27		1	126955500		[80]
Cotw72		1	127858114		[80]
ALVE_CAU021		1	129602884	No	[237]
New44		1	131129151		[80]
Cotw59		1	131205606		[80]
New7		1	134178691		[80]
Cotw68		1	142839720		[80]
ALVE_CAU017		1	143035486	No	[237]
ALVE-NSAC2		1	145339758		[80]
New40		1	152532430		[80]
Cotw73		1	154707214		[80]
Cotw69		1	157946035		[80]
ALVE_ros002	Unknown	1	158775708	No	[63]
ALVE_ros003	Complete	1	163248553	No	[63]
Cotw70		1	168930023		[80]
New37		1	172963412		[80]
ALVE_CAU013		1	182903121	No	[237]
Cotw71		1	183446398		[80]
New41		1	186593970		[80]

New4		1	187322258		[80]
ALVE-TYR	Complete	1	187921213	Tyr int4	[63]
ALVE_ros100		2	54009821	CNTNAP2 int14	[237]
ALVEB11		2	63525425		[236
ALVE_CAU003		2	71978681	No	[237]
ALVE_CAU006		2	104963623	No	[237]
ALVE_CAU007		2	113949658	No	[237]
ALVE-NSAC1	pol-env-LTR	2	120868843	No	[63]
ALVE_ros004	Complete	2	124432997	No	[63]
ALVE_ros005	LTR	2	142480536	No	[63]
ALVE_ros127	Complete	2	147349291	TSNARE1 int2	[237]
ALVE_CAU014		3	950253	FANCL int7	[237]
ALVE_CAU008		3	11026451	No	[237]
ALVE_CAU001		3	32243447	No	[237]
ALVE_CAU009		3	43153318	PDE10A int1	[237]
ALVE-NSAC3	Unknown	3	53639776	No	[63]
ALVE_ros006	Unknown	3	57337987	No	[63]
ALVE15	LTR	3	70384294	GRIK2 int16, involved in egg color	[63]
ALVE-NSAC5		3	72497167		[238]
ALVE_CAU010		3	76499348	No	[237]
ALVE_CAU002		3	89260091	CSMD1 int1	[237]
ALVE_CAU018		3	110009243	CD2AP int2	[237]
ALVEB10		4	28726730	Inside a CR1 element	[236]
ALVE_CAU019		4	42984321	GALNTL6 int4	[237]
ALVE_CAU004		4	54377551	LOC112532303 int5	[237]
ALVE_ros007	env-LTR	4	59843015	No	[63]

ALVE_ros008	Complete	4	62680158	No	[63]
ALVE_ros009	Unknown	4	71095932	No	[63]
ALVEB9		5	13230858		[236]
ALVE_CAU023		5	21052270	LRRC4C int1	[237]
ALVEB1		5	22536766	C5H11orf49 int3	[235]
ALVE-NSAC6		5	57020012		[238]
ALVE_CAU005	Complete	6	7270339	PCDH15 int27	[237]
ALVE9	pol-env-LTR	6	33153441	DOCK1 int35	[63]
ALVE4		6	33827722		[81]
ALVE_ros220		7	14856972	ZNF385B int2	[237]
ALVE_CAU015		8	10388286	No	[237]
ALVE-NSAC7	Complete	9	11714130	No	[63]
ALVE_ros010	Unknown	9	11871576	No	[63]
ALVE_CAU022		9	12011455	No	[237]
ALVE_ros240		9	12295680	LOC112533006 int4	[237]
ALVE-NSAC4		12	17574950		[238]
ALVEB6		14	9308658		[235]
ALVE8		20	1468850		[81]
ALVEB8		20	1762777		[235]
ALVE_ros273		20	3074677	PTPRT int7	[237]
ALVE3	Complete, lacks RT	20	10309347	HCK int6	[63]
ALVE_ros276	Complete	23	5645068	NT5C1A 3’UTR	[237]
ALVE_CAU020		Z	304564	No	[237]
ALVE21	Complete	Z	10681671	Notoriously linked to K gene	[63]
ALVE7		Z	14471852		[81]
